# The expression of the NPR1-dependent defense response pathway genes in *Persea americana* (Mill.) following infection with *Phytophthora cinnamomi*

**DOI:** 10.1186/s12870-023-04541-z

**Published:** 2023-11-08

**Authors:** Robert Backer, Sanushka Naidoo, Noëlani van den Berg

**Affiliations:** 1 Hans Merensky Chair in Avocado Research, University of Pretoria, Pretoria, South Africa; 2 Department of Biochemistry, Genetics and Microbiology, Faculty of Natural and Agricultural Sciences, University of Pretoria, Pretoria, South Africa; 3 Forestry and Agricultural Biotechnology Institute, Faculty of Natural and Agricultural Sciences, University of Pretoria, Pretoria, South Africa

**Keywords:** RNA-sequencing, NPR1, *Phytophthora cinnamomi*, *Persea americana*, Pathogenesis-related, Systemic acquired resistance, Salicylic acid

## Abstract

A plant’s defense against pathogens involves an extensive set of phytohormone regulated defense signaling pathways. The salicylic acid (SA)-signaling pathway is one of the most well-studied in plant defense. The bulk of SA-related defense gene expression and the subsequent establishment of systemic acquired resistance (SAR) is dependent on the nonexpressor of pathogenesis-related genes 1 (NPR1). Therefore, understanding the NPR1 pathway and all its associations has the potential to provide valuable insights into defense against pathogens. The causal agent of Phytophthora root rot (PRR), *Phytophthora cinnamomi,* is of particular importance to the avocado (*Persea americana*) industry, which encounters considerable economic losses on account of this pathogen each year. Furthermore, *P. cinnamomi* is a hemibiotrophic pathogen, suggesting that the SA-signaling pathway plays an essential role in the initial defense response. Therefore, the NPR1 pathway which regulates downstream SA-induced gene expression would be instrumental in defense against *P. cinnamomi*. Thus, we identified 92 NPR1 pathway-associated orthologs from the *P. americana* West Indian pure accession genome and interrogated their expression following *P. cinnamomi* inoculation, using RNA-sequencing data. In total, 64 and 51 NPR1 pathway-associated genes were temporally regulated in the partially resistant (Dusa®) and susceptible (R0.12) *P. americana* rootstocks, respectively. Furthermore, 42 NPR1 pathway-associated genes were differentially regulated when comparing Dusa® to R0.12. Although this study suggests that SAR was established successfully in both rootstocks, the evidence presented indicated that Dusa® suppressed SA-signaling more effectively following the induction of SAR. Additionally, contrary to Dusa®, data from R0.12 suggested a substantial lack of SA- and NPR1-related defense gene expression during some of the earliest time-points following *P. cinnamomi* inoculation. This study represents the most comprehensive investigation of the SA-induced, NPR1-dependent pathway in *P. americana* to date. Lastly, this work provides novel insights into the likely mechanisms governing *P. cinnamomi* resistance in *P. americana*.

**Supplementary Information:**

The online version contains supplementary material available at 10.1186/s12870-023-04541-z.

## Introduction

The cultivation of *Persea americana* (avocado), as with any crop, is adversely affected by the prevalence of pests and diseases, the most prominent being Phytophthora root rot (PRR). The causal agent, *Phytophthora cinnamomi* Rands is a hemibiotrophic oomycete which is difficult, if not impossible, to be eradicated from the environment due to an excessively broad-host range and persistent reproductive structures [1]. Control methods for PRR are a prominent focus in the agricultural industry, as avocado trade contributes significantly to the global economy, accounting for an estimated gross production value of $6.56 billion (constant 2014–2016, int. $) [2]. Rootstocks which are partially resistant to *P. cinnamomi*, such as the industry standard Dusa®, along with phosphite trunk injections and good orchard management practices are currently the best-known methods for limiting the impact of PRR in avocado orchards [3]. However, our limited understanding of the molecular defense mechanisms by which resistance is conferred has impeded the rate of new rootstock development, forcing a reliance on time-consuming *P. cinnamomi* resistance selection processes.

The phytohormone salicylic acid (SA) is an essential component of several plant defense responses [4–6]. For example, significant accumulation of SA at the initial site of infection is essential to the induction of the hypersensitive response (HR). Subsequently, the accumulation of SA in distal tissues initiates the establishment of systemic acquired resistance (SAR); a long-term, systemic defensive state initiated by SA-dependent gene expression following biotrophic/hemibiotrophic pathogen challenge [7, 8]. Intriguingly, SAR induced plants feature increased resistance to virtually all classes of pathogens, including viruses, bacteria, fungi, oomycetes and nematodes [8, 9]. Generally, SAR is defined by a substantial and sustained accumulation of a suite of antimicrobial pathogenesis-related (PR) proteins in tissues distal to the initial site of infection [5, 10, 11].

The induction of SAR is dependent on the nonexpressor of pathogenesis-related genes 1 (NPR1), a co-transcription factor known as the master regulator of defense responses [12–16]. Not only does SAR not establish in *Arabidopsis thaliana npr1* mutants, the induced expression of *PR1* and *PR5* is significantly decreased [12, 13]. Furthermore, the complementation using *NPR1* restores the wild-type *PR* gene expression, as well as the inducibility of SAR [14]. Thus, since its discovery, transgenic overexpression of *NPR1* has proven to increase disease resistance against a variety of pathogens across an expansive range of crops [17]. However, the complexity of NPR1-dependent gene expression requires a holistic view of all associated proteins.

Transcription factors are a central aspect of NPR1-dependent gene regulation. The promoters of typical NPR1-dependent genes, such as *PR1*, contain the SA-responsive *as-1-*like promoter element [18, 19]. Importantly, the TGACG-binding (TGA) transcription factor protein family associates with this promoter element and is required for SAR-related gene expression [18, 20, 21]. Furthermore, NPR1 and various TGA transcriptions factors interact directly, which ultimately increases their DNA-binding affinity [22–25]. However, TGA transcription factors have also been associated with negative regulatory promoter elements, suggesting that together, NPR1 and TGA transcription factors may also serve to suppress gene expression [24, 26].

Another essential, SA-responsive, transcription factor family are the WRKYs [27, 28]. Though these transcription factors were initially suggested to suppress the expression of SAR-related genes during non-stress conditions, many have since been implicated in positive regulation of defense signaling [27–31]. The WRKY transcription factor specific W-box *cis*-elements are common in many SAR-related genes, including *isochorismate synthase 1* (ICS1), *TL1-binding transcription factor (TBF1)*, *PR1* and even *NPR1* itself [27, 32–36]. It was also shown that in certain situations, TGAs, WRKYs and NPR1 might all work together to regulate SA-dependent gene expression [37]. Thus, various transcription factors serve to extend the influence of NPR1 on SA-dependent gene expression, contributing to its broad regulatory effect on several types of pathogens.

Although the expression of *NPR1* is itself SA-responsive, post-transcriptional modification of NPR1 seems to be at least as important as increased expression [17]. The most extensively studied NPR1 post-translational change happens within the cytoplasm; here, during non-stress conditions, NPR1 exists as an oligomer [38]. Following the SA-induced oxidative burst associated with pathogenic stress, and the increased production of reducing agents, NPR1 is monomerized [38]. The action of thioredoxins (TRXs), in particular, are responsible for the reduction of NPR1^Cys156^, resulting in its monomerization [38, 39]. By contrast, *S*-nitrosoglutathione (GSNO) promotes the existence of NPR1 as an oligomer [39]. Interestingly, a class III type alcohol dehydrogenase (ADH), *S*-nitrosoglutathione reductase (GSNOR), reduces the amount of available GSNO [40]. Moreover, expression of the gene encoding for GSNOR in *A. thaliana* is induced by SA and essential to the establishment of SAR [41–43]. Thus, taken together, the actions of TRX and GSNOR would reduce the potential for NPR1 to exist as an oligomer and in so doing contribute to the establishment of SAR.

Several protein kinases have also been described in the post-translational regulation of NPR1 activity [44–46]. Interestingly, the NPR1 residue Ser589, which is essential as a part of the nuclear localization signal (NLS2), is phosphorylated by the sucrose non-fermenting 1 (SNF1)-related protein kinase 2 (SRK2C) protein [45, 47]. Furthermore, *SRK2C* is expressed in response to SA-independent systemic signals and thus, presumably, plays a role in the nuclear import of NPR1 in distal tissues, where SA concentration is lower [45, 46]. Similar to SRK2C, CBL-interacting serine/threonine-protein kinase 11 (CIPK11) interacts with and phosphorylates the C-terminal region of NPR1 [46]. In *Arabidopsis* this modification ultimately leads to upregulated expression of *WRKY38* and *WRKY62* in response to *Pseudomonas syringae* pv. *tomato* DC3000 [46].

Moreover, phosphorylation of Ser11/15 and Ser55/59 reinforces sumoylation of NPR1 by the small ubiquitin-like modifier 3 (SUMO3), a positive regulator of SA-induced gene expression [29, 48, 49]. Overall, the sumoylation of NPR1 decreases its interaction with WRKYs, while increasing interaction with TGAs [49]. Furthermore, sumoylation of NPR1 leads to increased phosphorylation of Ser11/15, reinforcing defense gene expression, followed by ubiquitinylation and subsequent proteasome-mediated turnover of spent NPR1 [49, 50]. Notably, the turnover of NPR1 completes SAR induction, as inherently unstable co-transcription factors likely cannot maintain peak gene expression without being replaced continuously [50–52]. However, neither CUL3 or E3-ligases, which ubiquitinylate NPR1, have been shown to interact with NPR1 directly and therefore likely require a substrate adapter [19, 50, 53, 54].

Interestingly, NPR1 increases the expression of several protein secretory pathway genes, likely to ensure correct protein processing in response to increased PR protein production [34, 55]. These genes all have a common *TL1 cis-*element within their promoters which are bound by the heat stress transcription factor, TBF1 [34, 55]. Although *A. thaliana tbf1* mutants do not display decreased *PR1* transcript or protein levels, the secretion of PR1 into the apoplast is substantially reduced [34]. Interestingly, both *tbf1* and *npr1-1* mutants presented with a decreased expression of *luminal binding protein 2* (*BiP2*) and *calreticulin* 3 (*CRT3*). These observations, together with the presence of the appropriate promoter *cis*-elements, suggest that the expression of *NPR1* and *TBF1* is likely co-regulated [34]

Additionally, SA-responsive negative regulators, such as the NIM(NPR1)-interacting (NIMIN) proteins, are another key component of NPR1-dependent gene expression [56, 57]. However, the effect of NIMINs is not absolute, and instead, these proteins impact the timing of gene expression [58]. Furthermore, proteins such as NPR3 and NPR4, which serve redundant negative regulatory roles, oppose to the function of NPR1 [19]. These *bone fide* SA receptors associate with several TGAs and the promoters of SA-inducible genes, preventing expression in the absence of SA [19]. Interestingly, the expression of *histone deacetylase 19* (*HDAC19*), a negative regulator of SAR, is NPR1 and SA dependent [59]. Moreover, repression of *PR1* and *PR2* is, at least in part, regulated by HDAC19, which associates with and deacetylates their respective promoters, limiting expression during uninduced conditions [59]. These studies highlight another critical aspect of SA-inducible, NPR1-dependent gene expression i.e., timing.

The correct timing of defense responses underpins their effectiveness and prevents potential fitness loss due to unnecessary, uninduced defense gene expression. Here, priming forms an integral aspect of SAR, allowing for an earlier, stronger, and thus more effective defense response during subsequent pathogen challenge [60, 61]. In *A. thaliana* the expression of NPR1-dependent, pathogen-responsive *mitogen-activated protein kinase 3 (MPK3)* and *MPK6* have been implicated in the priming of SA-induced defense responses [62]. The accumulation of inactive, unphosphorylated MPK3/6 and their transcripts allows for quicker signal transduction and subsequent responses in reaction to pathogens [62–64]. Furthermore, expression of the circadian clock genes*, timing of cab2 expression 1* (*TOC1*) and its antagonist *late elongated hypocotyl* (*LHY*), is NPR1-dependent [65]. Together, TOC1 and LHY control the balance of growth and defense throughout the day, prioritizing defense in the morning when pathogen pressure is at its peak [65–68].

The existence of complex defense mechanisms in plants have enabled them to combat the virulence strategies employed by various pathogens [69]. Ultimately, host–pathogen interactions can be defined, towards either extreme, as compatible/susceptible or incompatible/resistant [70, 71]. However, host–pathogen interactions are far from binary, given their complexity, and should instead be described on a spectrum, ranging from entirely susceptible to fully resistant. Understanding, at least some of, this complexity may provide insights which could aid in breeding crops for increased pathogen resistance.

As evidenced, a multitude of studies have investigated components of the NPR1-dependent defense response pathway genes during plant-pathogen interactions, including several specifically addressing the interaction between plants and *Phytophthora* spp. [72–78]. However, to the best of our knowledge, none have explored a complete representation of this pathway, especially during *Phytophthora*-plant interactions. Previously, we described five *NPR1-like* genes in *Persea americana*, three of which are likely to partake in the defense response against *P. cinnamomi* [79]. However, attempting to understand the regulation of the NPR1 pathway-associated genes in totality seems sensible given the intricacy of NPR1-dependent gene expression. We believe that regulation of NPR1 pathway-associated genes in *P. americana* will, to some extent, resemble expectations based on model systems such as *Arabidopsis*, following pathogen challenge. Furthermore, we expect to see notable differences in the regulation of several NPR1 pathway-associated genes between susceptible and partially resistant *P. americana* rootstocks in response to *P. cinnamomi* inoculation. In the current study, we endeavored to identify and partially characterize a wide variety of NPR1 pathway-associated genes from the *P. americana* West Indian (WI) pure accession genome. Using RNA-sequencing we compared the expression of 92 unique *P. americana* NPR1 pathway-associated genes from both the *P. cinnamomi* susceptible (R0.12) and partially resistant (Dusa®) rootstocks, following inoculation. We described the response of both rootstocks to *P. cinnamomi* inoculation across four time points. Additionally, we compared the expression of these genes between Dusa® and R0.12. Overall, the expression of most NPR1 pathway-associated genes responded as expected based on the literature, indicating activation of this pathway within 24 h post-inoculation (hpi) and the establishment of SAR by 120 hpi. However, the response in Dusa® appeared to be more robust with more NPR1 pathway-associated genes displaying differential expression at several of the investigated time points. The most apparent differences between Dusa® and R0.12 were observed at 12 and 24 hpi. Thus, this study provides the first evidence of significant regulatory differences regarding the expression of NPR1 pathway-associated genes in response to challenge by *P. cinnamomi*.

## Materials and methods

### Plant material

Approximately two-year-old clonal Dusa® and R0.12 plantlets, which are partially resistant or susceptible to *P. cinnamomi,* respectively, were provided by Westfalia™ Innovation and Technology (Tzaneen, ZAF). Plantlets were acclimatized in a temperature-controlled glasshouse at 25 °C for 2 weeks and watered when the surface of the media was dry to the touch. Plantlets were transplanted into a 1:1 perlite vermiculite medium following the removal of the nurse seed, a remnant of the Frolich technique [80], and left to acclimatize for an additional 2 weeks.

### *P. cinnamomi* infection trial

The *P. cinnamomi* isolate GKB4 was obtained from the Avocado Research Programme Culture Collection (Pretoria, ZAF). Virulence of the isolate was recovered through apple inoculation followed by single hyphal tip re-isolation [81]. Zoospores were produced for the inoculation of *P. americana,* as described in [82]. Plantlets were randomly assigned to either the treatment or control group. The treatment group was inoculated by submerging the roots in a zoospore suspension (1.4 × 10^5^ zoospore.ml^−1^), while those of the control group were mock-inoculated by immersion in dH_2_O (uninoculated control), both treatments were done at midday. Plantlets were replanted in a 1:1 perlite vermiculite medium 2 h after inoculation. The treatment group consisted of three biological replicates with three plantlets per replicate for both Dusa® and R0.12. Samples were collected at 6, 12, 24 and 120 hpi based on the proposed timeframes for the biotrophic and necrotrophic life stages of *P. cinnamomi* during the infection of *P. americana* [3]. Due to material limitations the control group was only harvested at 24 hpi. Harvested roots were snap-frozen in liquid nitrogen and stored at -80 °C. Biological replicates were homogenized using the IKA® Tube Mill control (IKA®, Staufen, DEU) until a fine consistency was attained.

### RNA-sequencing

Total RNA was extracted from homogenized plant material using a modified version of the CTAB extraction method [83]. The chloroform: isoamyl alcohol step was repeated four to six times until the volume of the interphase diminished, and the supernatant was clear. Samples were treated with DNase I (Fermentas Inc., Vilnius, LTU) and purified using the Qiagen RNeasy clean up kit (Qiagen Inc., Valencia, California, USA). Samples were resuspended in diethylpyrocarbonate (DEPC) treated water containing 30 U.ml^−1^ RiboLock RNase Inhibitor (Thermo Fisher Scientific Inc., Leicestershire, GBR). Conventional PCR using intron-spanning flavone-3-hydroxylase (F3H) primers was used to confirm the absence of DNA contamination [84]. RNA concentration and purity were assessed using the NanoDrop® ND-1000 spectrophotometer (Nanodrop Technologies Inc., Montchanin, Delaware, USA). RNA integrity was evaluated on 2% TAE agarose gel under non-denaturing conditions as well as capillary electrophoresis on the Agilent 2100 Bioanalyzer (Agilent Technologies Inc., Santa Clara, California, USA). All inoculated and uninoculated samples from both Dusa® and R0.12 were submitted for paired-end sequencing to at least 80 × coverage on the Illumina® HiSeq™ PE150 platform (Novogene Corporation Inc., Chula Vista, California, USA).

### Quality control and read mapping

Read quality, both prior to and following trimming was assessed using FastQC v0.11.9 [85] and subsequently summarized using MultiQC [86]. Raw data were trimmed of low quality bases and Illumina sequencing adapters using Trimmomatic v0.39 [87]. RNA-seq reads were then mapped to a concatenated *P. cinnamomi* GKB4 [88] and *P. americana* WI pure accession genome (Avocado Genome Consortium, Article in preparation), using HISAT2 v2.0.6 [89]. Portcullis v1.2.0 [90] was used to obtain high-confidence splice junctions which served as additional input during a second round of read mapping in HISAT2.

### Differential gene expressions analyses

Gene level read counts for each sample library were quantified using the *P. americana* WI pure accession genome annotation as reference, thereby excluding all reads that mapped to the *P. cinnamomi* GKB4 genome, using featureCounts v2.0.1 [91]. Count tables were input in R 4.0.4 [92] and subject to analyses offered by DeSeq2 v1.32.0 [93]. Sample library read data were filtered during analyses to remove individual observations with fewer than 10 reads, furthermore any transcripts with no read data were removed entirely. Time course expression analyses for either R0.12 or Dusa® were performed by designating sample libraries representing uninoculated samples for either rootstock as the references for the respective rootstock’s inoculated sample libraries. To compare the expression of genes within uninoculated and each of the time point sample libraries between R0.12 and Dusa®, R0.12 sample libraries were set as the reference. The Wald test was used to identify differentially expressed genes (DEGs) and the Benjamini-Hochberg (BH) false discovery rate (FDR) method was used for multiple hypotheses testing correction. DEGs were defined as significant using an FDR cutoff (adjusted *p*-value; p-adj) of less than 0.05 a log_2_(fold change; log_2_FC), of more than 0.58 (upregulated) and less than -0.58 (downregulated), representing a 50% increase or decrease in expression, respectively. Finally, DEGs with a baseMean of less than 20 were excluded from further analyses. The FDR cutoff chosen for this study was intended to capture subtler changes in the expression of transcription factors, as small changes may have disproportionately large effects on downstream gene expression. Meanwhile, the baseMean cutoff was intended to limit the inclusion of misleading observations with low read counts.

### Candidate gene identification and annotation

**Fig. 1:**
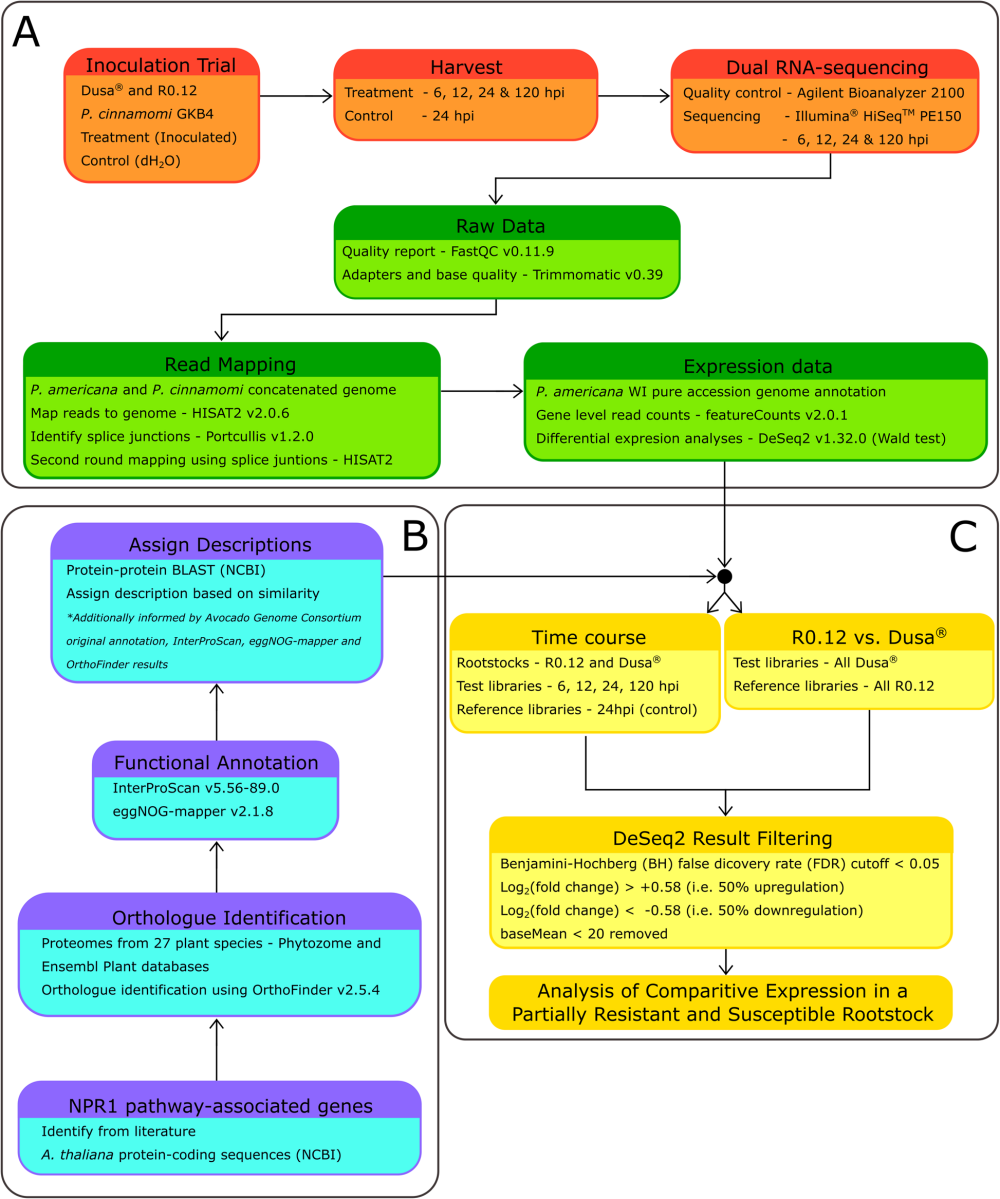
Visual representation of workflow used to ultimately determine differences in the expression of NPR1 pathway-associated genes in *Persea americana*. **A** Plantlets from two rootstocks were either inoculated with *Phytophthora cinnamomi* or dH_2_O. RNA-sequencing was performed on libraries derived from the 24 hpi control and 6, 12, 24 and 120 hpi inoculated samples for each rootstock. Raw data were processed and used for differential gene expression analyses. **B** A thorough search of the literature identified NPR1 pathway-associated genes [17]. Orthologues of NPR1 pathway-associated genes were identified in the *P. americana* genome after which functional annotation and protein–protein BLAST was used to complement existing annotations and assign final descriptors. **C** Two analyses were conducted in DeSeq2 – the first set the control sample libraries (uninoculated) as the reference to analyze changes in expression, over time, in each of the rootstocks. Secondly, all libraries for R0.12, including the control library, were set as references for the corresponding Dusa® libraries to determine differences in expression within each time point, between rootstocks. The Wald test was used to identify differentially expressed genes (DEGs) and the Benjamini-Hochberg (BH) false discovery rate (FDR) method was used for multiple hypotheses testing correction

Proteins that were identified as essential to the establishment of SAR or known to be NPR1-dependent were determined from the literature [17]. Corresponding *A. thaliana* protein-coding sequences were obtained online from NCBI (http://www.ncbi.nlm.nih.gov/) (Table 1). Twenty seven representative plant proteomes were obtained from the Ensembl Plants database (https://plants.ensembl.org/index.html) and Phytozome (https://phytozome-next.jgi.doe.gov/; Table S1) and used for putative ortholog identification, together with the primary transcript predicted peptides from *P. americana,* in OrthoFinder v2.5.4 [94]. All *P. americana* orthologs corresponding to the *A. thaliana* proteins identified previously (Table 1) were used in further analyses. InterProScan protein domain identifier v5.56–89.0 [95] was used to identify conserved domains while eggNOG-mapper v2.1.8 [96, 97] was used to complement the functional annotation. Finally, the identified *P. americana* orthologs sequences were subjected to a protein–protein BLAST (BLASTp) in CLC Main Workbench v21.0.3 (CLC Bio, Qiagen® Inc., Hilden, DEU) using the NCBI blast servers (https://blast.ncbi.nlm.nih.gov/Blast.cgi). The combined data were used to manually curate annotations for the final list of the *P. americana* NPR1-dependent defense response pathway proteins and assign descriptors. The experimental design and workflow for this study were summarized visually for additional reference (Fig. 1).

**Table 1:** NPR1 associated sequences from *A. thaliana* used to identify similar sequences from the *Persea americana*

Gene	Protein	Accession Number
*BiP2*	Heat shock protein 70 (Hsp 70) family protein	[Genbank: NM_180788]
*CIPK11/PKS5*	CBL-interacting serine/threonine-protein kinase 11	[Genbank: NM_128589]
*CRT3*	Calreticulin	[Genbank: NM_100718]
*CUL3A*	Cullin-3A	[Genbank: NM_102447]
*CUL3B*	Cullin-3B	[Genbank: NM_001334418]
*DAD1*	Phospholipase A(1) DAD1, chloroplastic	[Genbank: NM_001337097]
*GSNOR1*	*S*-(hydroxymethyl)glutathione dehydrogenase	[Genbank: NM_123761]
*HDAC19*	Histone deacetylase 19	[Genbank: NM_119974]
*HSFB1/TBF1*	Heat stress transcription factor B-1	[Genbank: NM_119862]
*ICS1*	Isochorismate synthase 1, chloroplastic	[Genbank: AY056055]
*LHY*	Protein LHY	[Genbank: NM_099988]
*MPK3*	Mitogen-activated protein kinase 3	[Genbank: NM_114433]
*MPK6*	Mitogen-activated protein kinase 6	[Genbank: NM_129941]
*NIMIN-1*	Protein NIM1-INTERACTING 1	[Genbank: AJ250184]
*NIMIN-2*	Protein NIM1-INTERACTING 2	[Genbank: AJ250185]
*NIMIN-3*	Protein NIM1-INTERACTING 3	[Genbank: AJ250186]
*PR1*	Pathogenesis-related protein 1	[Genbank: NM_127025]
*PR2/BGL2*	Beta-1,3-glucanase 2	[Genbank: NM_001339849]
*PR5*	Pathogenesis-related protein 5	[Genbank: NM_106161]
*SARD1*	Protein SAR DEFICIENT 1	[Genbank: NM_106040]
*Sec61*α	Protein transport protein Sec61 subunit alpha	[Genbank: AY093047]
*SRK2C/SnRK2.8*	Serine/threonine-protein kinase SRK2C	[Genbank: NM_001084370]
*SUMO3*	Small ubiquitin-related modifier 3	[Genbank: NM_001345118]
*TGA2*	Transcription factor TGA2	[Genbank: EF470791]
*TGA3*	Transcription factor TGA3	[Genbank: NM_102057]
*TGA4*	Transcription factor TGA4	[Genbank: NM_121041]
*TGA5*	Transcription factor TGA5	[Genbank: NM_203016]
*TGA6*	Transcription factor TGA6	[Genbank: NM_202564]
*TGA7*	Transcription factor TGA7	[Genbank: NM_106441]
*TOC1*	Timing of CAB expression 1 protein	[Genbank: AF272039]
*TRX3*	Thioredoxin H3	[Genbank: NM_123664]
*TRX5*	Thioredoxin H5	[Genbank: NM_103588]
*WRKY18*	WRKY transcription factor 18	[Genbank: NM_119329]
*WRKY29*	WRKY transcription factor 29	[Genbank: NM_118486]
*WRKY38*	WRKY transcription factor 38	[Genbank: NM_122163]
*WRKY40*	WRKY transcription factor 40	[Genbank: NM_106732]
*WRKY53*	WRKY transcription factor 53	[Genbank: NM_118512]
*WRKY6*	WRKY transcription factor 6	[Genbank: AF331713]
*WRKY60*	WRKY transcription factor 60	[Genbank: NM_128058]
*WRKY62*	WRKY transcription factor 62	[Genbank: NM_120268]
*WRKY70*	WRKY transcription factor 70	[Genbank: NM_115498]

## Results

### RNA-seq data analysis

RNA-sequencing of *P. americana* sample libraries produced a total of 4 515 906 733 paired-end reads following trimming. The susceptible (R0.12) sample libraries accounted for approximately 44% of the total reads while the partially resistant (Dusa®) sample libraries accounted for approximately 56%. Approximately 82% and 86% of R0.12 and Dusa® control sample library reads mapped back to the *P. americana* genome, respectively. Whereas 72% and 80% mapped back in the *P. cinnamomi* inoculated R0.12 and Dusa® samples. A complete report for the data included in this study was recently published [98].

### Identification and annotation of NPR1 pathway-associated genes

Using OrthoFinder, we identified 89 unique *P. americana* orthologs of the *A. thaliana* NPR1-dependent defense response pathway (Table 1), representing all query proteins except for *Arabidopsis SUMO3* (*AtSUMO3*). BLASTp using *AtSUMO3* to query a local BLAST database of *P. americana* proteins identified three proteins with high similarity (Expect value (E) cutoff of 1.0e^−10^). The final list of 92 *P. americana* NPR1-dependent defense response pathway proteins were manually curated using a combined approach to arrive at putative functional descriptions (Table S2). InterProScan successfully assigned functional domains to 97.8% of the 92 putative proteins. Meanwhile, eggNOG-mapper could assign functional domains and protein families to 92.4%. BLASTp analysis revealed that putative *P. americana* NPR1 pathway-associated proteins are most like orthologs in *Cinnamomum micranthum*, which accounted for 69 (75.0%) of the top-scoring hits. Considering the top scoring hits to all 92 putative proteins, the average percentage identity was 92.1% while E-value averaged 1.62e^−20^ (Table S3).

### Expression of NPR1 pathway-associated genes

**Fig. 2:**
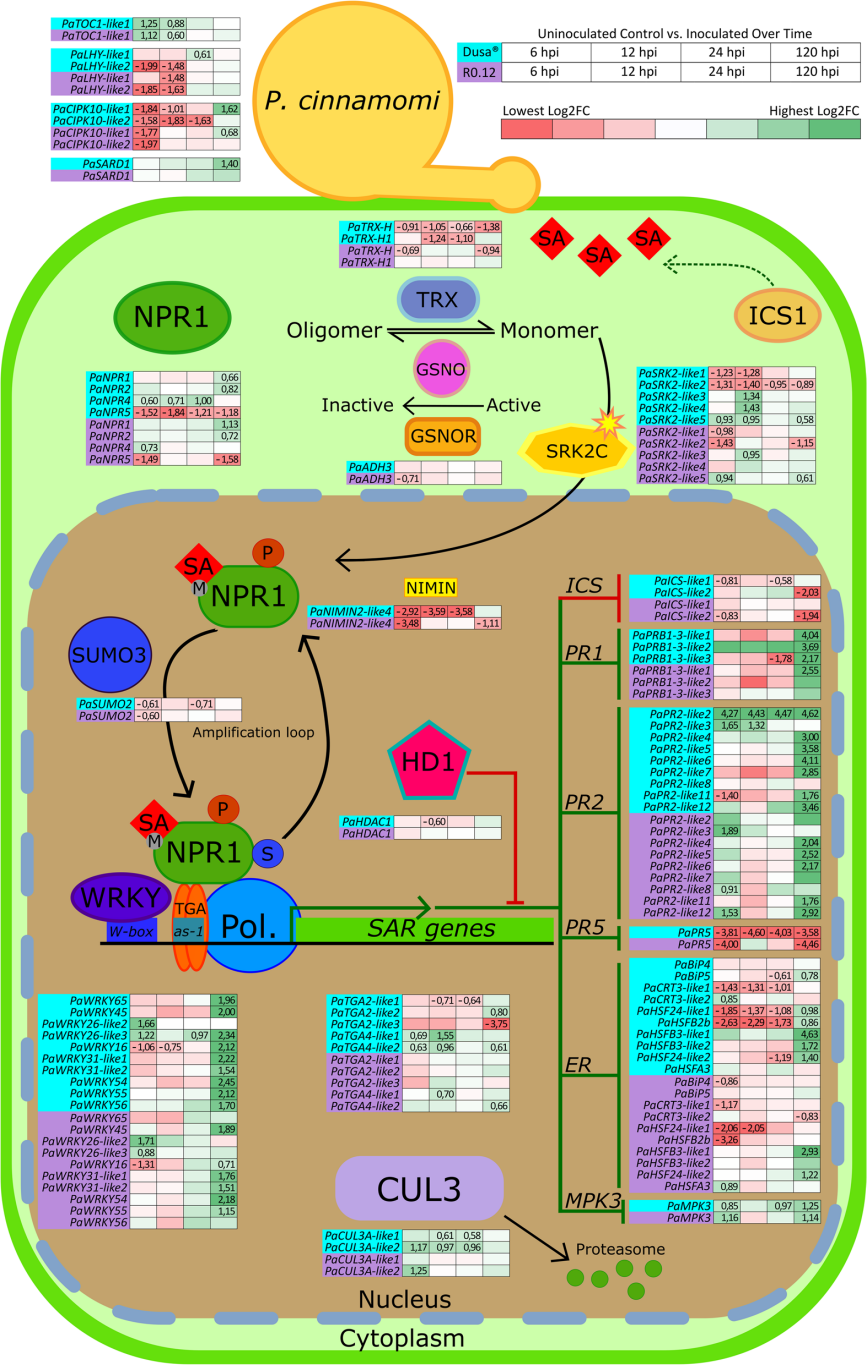
Visual representation of NPR1 pathway-associated differential gene expression in *Persea americana* during *Phytophthora cinnamomi* challenge. Analyses were conducted over time using *P. cinnamomi* inoculated sample libraries harvested at 6, 12, 24 and 120 h post-inoculation (hpi), in either the partially resistant (Dusa®) or susceptible (R0.12) *Persea americana* rootstock. Rootstock representative uninoculated control sample libraries, which were harvested at 24 hpi, were used as the reference*.* Gradient colored blocks indicate differential gene expression where red signifies downregulation (log_2_(fold change; log_2_FC) < -0.58) and green signifies upregulation (log_2_FC > 0.58) for a given gene across all observations, within a given rootstock. Gene symbols are indicated to the left of the expression data for each gene; here gene symbols for Dusa® are backed by a blue color, while for R0.12 gene symbols are backed in purple. Only values which were significantly up- or downregulated during at least one time point, in either rootstock, are visible and were determined using a false discovery rate (FDR) cutoff (adjusted *p*-value; p-adj) of less than 0.05. Genes for which expression data are not indicated within the working model were placed there for simplicity and do not imply function outside of the pathway. For more detailed information on each gene, including ones not included in this figure, please refer to Supplementary table S4. The graphical representation above, which summarizes the NPR1 pathway, was adapted from Backer et al*.* (2019). Abbreviations: Pol. (polymerase), P (phosphorylated), S (sumoylated)

**Fig. 3:**
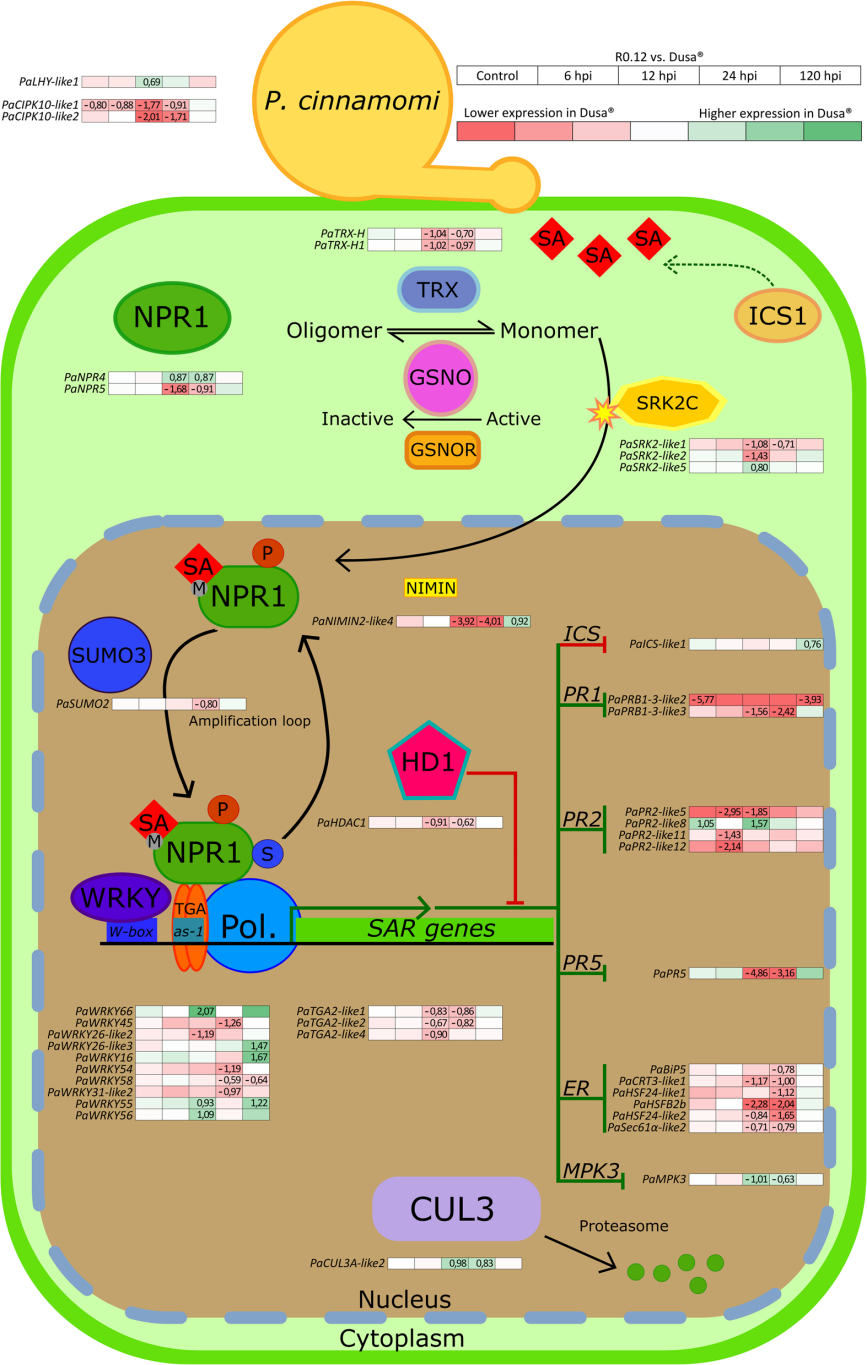
Visual representation of NPR1 pathway-associated gene expression during *Phytophthora cinnamomi* challenge. Analyses were conducted by comparing all sample libraries from the partially resistant *Persea americana* rootstock Dusa® to the corresponding sample libraries in the susceptible rootstock R0.12. Sample libraries included in this comparison were the uninoculated control, which was harvested at 24 h post-inoculation (hpi), and *P. cinnamomi* inoculated samples harvested at 6, 12, 24 and 120 hpi. Gradient colored blocks indicate differential gene expression where red signifies downregulation (log_2_(fold change; log_2_FC) < -0.58) in Dusa® when compared to R0.12, and green signifies upregulation (log_2_FC > 0.58) for a given gene across all observations. Gene symbols are indicated to the left of the expression data for each gene. Only values which were significantly up- or downregulated for at least one comparison are visible and were determined using a false discovery rate (FDR) cutoff (adjusted *p*-value; p-adj) of less than 0.05. Genes for which expression data are not indicated within the working model were placed there for simplicity and do not imply function outside of the pathway. For more detailed information on each gene, including ones not included in this figure, please refer to Supplementary table S5. The graphical representation above, which summarizes the NPR1 pathway, was adapted from Backer et al*.* (2019). Abbreviations: Pol. (polymerase), P (phosphorylated), S (sumoylated)

We examined the expression of 92 NPR1 pathway-associated genes in both the *P. cinnamomi* susceptible (R0.12) and partially resistant (Dusa®) *P. americana* rootstocks. The expression data from four *P. cinnamomi* inoculated time point sample libraries (6, 12, 24 and 120 hpi) were compared within each rootstock using the corresponding rootstock uninoculated control libraries as reference. In separate analyses the expression data were also used to compare expression between Dusa® and R0.12 sample libraries by setting the latter rootstock as the reference for expression in the former. Four genes were excluded from further analyses as no expression data were available, while 10 were excluded based on low average expression values (baseMean < 20). Significant DEGs for both analyses were organized into heatmaps and displayed in a schematic representation to contextualize the role of each gene within the NPR1-dependant pathway (Figs. 2 and 3). Of the remaining 78 NPR1 pathway-associated genes, 64 displayed significant differential expression during at least one time point (log_2_FC > 0.58 |< -0.58, adjusted *p*-value (FDR) < 0.05), when compared to the control in Dusa®. Meanwhile, in R0.12 only 51 genes displayed significant differential expression during at least one time point. When comparing each of the sample conditions between rootstocks, three genes displayed significant differences between uninoculated libraries, four at 6 hpi, 29 at 12 hpi, 25 at 24 hpi and seven at 120 hpi (Fig. 3). The log_2_FC, adjusted *p*-values and standard error of log_2_FC for all 92 NPR1 pathway-associated genes, in both rootstocks and for both analyses, can be found in Supplementary tables S4 and S5.

#### *PaNPR1-like* genes

Five putative *PaNPR1-like* genes were identified in *P. americana*, in keeping with a previous study [79]. Significant differences in expression were observed in both rootstocks, over time, for four of these genes, *PaNPR1*, *PaNPR2*, *PaNPR4*, and *PaNPR5* (Fig. 2). The expression of *PaNPR1* and *PaNPR2*, in either rootstock, did not differ significantly from uninoculated control at 6, 12 and 24 hpi. However, expression of these genes was significantly upregulated at 120 hpi in both rootstocks, although their upregulation at this point was not substantial. By contrast, *PaNPR4* was significantly upregulated at 6, 12 and 24 hpi in Dusa®, with slightly elevated log_2_FC values observed at each successive early time point, concluding with a return to baseline at 120 hpi. In R0.12 *PaNPR4* was only significantly upregulated at 6 hpi with a return to baseline observed for all successive time points. The expression of *PaNPR5* was significantly downregulated at all time points in Dusa®, while in R0.12 downregulation was only observed at 6 and 120 hpi. Overall, the expression of all four differentially expressed *PaNPR1-like* genes displayed similar trends when compared to their respective controls at 6 and 120 hpi, with clear deviations becoming apparent at 12 and 24 hpi. These observations were supported by significant differences in the expression of *PaNPR4* and *PaNPR5* at 12 and 24 hpi, when comparing expression between rootstocks (Fig. 3). The expression of *PaNPR4* was significantly higher in Dusa® at 12 and 24 hpi, while remaining comparable in all other sample libraries (uninoculated control, 6 and 120 hpi). In comparison, *PaNPR5* expression was significantly lower in Dusa® at 12 and 24 hpi.

#### *PaNPR1-like* post-translational modification related genes

Several genes that encode for proteins involved in post-translation modification of NPR1-like proteins showed statistically significant differences in expression. Two *AtTRX-lik*e orthologs were identified in *P. americana*, *PaTRX-H* and *PaTRX-H1*. The first of these, *PaTRX-H*, was significantly downregulated across all time points in Dusa® when compared to uninoculated control, while in R0.12 significant downregulation was only seen at 6 and 120 hpi (Fig. 2). The second ortholog, *PaTRX-H1* only displayed significant downregulation in Dusa®, at 12 and 24 hpi, and not in R0.12. Again, like the expression data for *PaNPR1-like* genes, *PaTRX*’s seemed to differ primarily at 12 and 24 hpi, showing significantly lower expression in Dusa® when compared to R0.12 (Fig. 3). Only one gene in *P. americana*, *PaADH3,* was identified as an ortholog of *AtGSNOR1* and it was significantly downregulated at 6 hpi in R0.12 (Fig. 2). However, no significant differences were observed when comparing the expression in Dusa® and R0.12 across any of the sample libraries.

Five putative orthologs of the serine/threonine-protein kinase encoding gene *AtSRK2C* namely, *PaSRK2-like1*, *PaSRK2-like2*, *PaSRK2-like3*, *PaSRK2-like4*, and *PaSRK2-like5* were identified in *P. americana*. All five showed significant differences in expression for Dusa® sample libraries, while only four were significantly different from the control in R0.12 (Fig. 2). *PaSRK2-like1* was significantly downregulated at 6 and 12 hpi in Dusa*®* and at 6 hpi in R0.12. In Dusa®, the expression of *PaSRK2-like2* was significantly lower when compared to control across all time points, but only at 6 and 120 hpi in R0.12. By contrast, *PaSRK2-like3* and *PaSRK2-like4* were significantly upregulated in Dusa® at 12 hpi. However, in R0.12 only *PaSRK2-like3*, and not *PaSRK2-like4*, was significantly upregulated at 12 hpi. *PaSRK2-like5* was significantly upregulated, most noticeably, at 6 and 12 hpi in Dusa® and 6 hpi in R0.12, barely meeting the minimum cutoff for log_2_FC in both rootstocks at 120-hpi. When comparing R0.12 and Dusa® three *PaSRK2-like* orthologs showed significant differences in expression (Fig. 3). The expression of *PaSRK-like1* at 12 and 24 hpi, and *PaSRK2-like2* at 12 hpi was significantly lower in Dusa®. By contrast, significantly more *PaSRK-like5* was expressed in Dusa® at 12 hpi when compared to R0.12.

Additional putative serine/threonine-protein kinase encoding genes, *PaCIPK10-like1* and *PaCIPK10-like2*, were identified as orthologs of *AtCIPK11*. In R0.12, expression of these genes was significantly lower than uninoculated control at 6 hpi returning to baseline at 12 and 24 hpi (Fig. 2). At 120 hpi, the expression of *PaCIPK10-like1* increased slightly above the log_2_FC cutoff value, with statistically significant support. Similarly, both genes were significantly downregulated in Dusa® at 6 hpi, however the expression of *PaCIPK10-like1* remained significantly lower when compared to the uninoculated control at 12 hpi, returning to baseline levels at 24 hpi and increasing substantially by 120 hpi. *PaCIPK10-like2* remained significantly downregulated throughout the early time points (6, 12 and 24 hpi) in Dusa®, only returning to baseline levels at 120 hpi. Interestingly, the expression of *PaCIPK10-like1* was significantly lower in Dusa® for both the early time point and the uninoculated control samples, when compared against R0.12 (Fig. 3). Likewise, *PaCIPK10-like2* expression was lower in Dusa® at 12 and 24 hpi while expression was comparable for all other sample libraries.

The gene which encodes for an NPR1-interacting protein in *A. thaliana*, *AtNIMIN-2*, was used to identify five putative orthologs in *P. americana*. However, only one of these, *PaNIMIN2-like4* met the average sample library expression criteria set forward by this study (baseMean > 20). Expression of this gene was significantly downregulated in both the susceptible and partially resistant rootstocks at 6 hpi (Fig. 2). In Dusa® this downregulation continued into 12 and 24 hpi, while in R0.12 expression returned to baseline at these time points. At 120 hpi, *PaNIMIN2-like4* expression was significantly downregulated in R0.12, but not Dusa®. When comparing the expression of *PaNIMIN2-like4* between these two rootstocks directly, the expression was comparable in the uninoculated control and 6 hpi samples (Fig. 3). However, at 12 and 24 hpi, expression was significantly lower in Dusa® when compared to R0.12. Conversely, at 120 hpi the abundance of *PaNIMIN2-like4* was significantly higher in Dusa®. Interestingly, the statistically significant log_2_FC’s for *PaNIMIN2-like4* were substantial when viewed against the differentially expressed genes reported on thus far.

Curiously, no *AtSUMO3* orthologs were identified using OrthoFinder, instead BLASTp was used to identify three similar putative proteins in *P. americana*—*PaSUMO1-like1, PaSUMO1-like2* and *PaSUMO2*. The expression of *PaSUMO1-like1* and *PaSUMO1-like2* did not meet any of the criteria for significant expression differences at any time point, in either rootstock. *PaSUMO2* was considered significantly downregulated at 6 and 24 hpi in Dusa® and at 6 hpi in R0.12 (Fig. 2). Notably, downregulation of this gene was minimal when compared to the control in each rootstock. When comparing the rootstocks however, the expression of *PaSUMO2* was significantly lower in Dusa® than in R0.12, at 24 hpi (Fig. 3). Expression of this gene was comparable between the rootstocks when considering comparisons between all other sample libraries.

Two orthologs of *AtCUL3A* were identified in *P. americana*, *PaCUL3A-like1* and *PaCUL3A-like2.* Observed changes in the expression of these genes at 6 hpi in both rootstocks revealed no differences for *PaCUL3A-like1*, but significant upregulation of *PaCUL3A-like2* in both Dusa® and R0.12 (Fig. 2). Interestingly, *PaCUL3A-like2* expression remained upregulated at 12 and 24 hpi in Dusa® but not R0.12. In addition, the expression of *PaCUL3A-like1* increased slightly, yet significantly, at 12 and 24 hpi in Dusa®, but not R0.12. It was thus unsurprising to note significantly higher expression of *PaCUL3A-like2* in Dusa® at 12 and 24 hpi when compared to R0.12 (Fig. 3).

#### Transcription factor genes

Transcription factors are essential to the coordinated expression of SAR-related genes [17]. Although 15 *AtWRKY-like* orthologs were identified in *P. americana*, only 10 were found to be significantly differentially expressed in response to inoculation with *P. cinnamomi*, when considering both rootstocks (Fig. 2). Interestingly, seven *PaWRKY-like* genes in Dusa®, and five in R0.12 were exclusively upregulated at 120 hpi when compared to their respective uninoculated controls. Significant upregulation of *PaWRKY65* and *PaWRKY56* at 120 hpi was only observed in Dusa®. When comparing Dusa® and R0.12 three of these genes, *PaWRKY45*, *PaWRKY54*, and *PaWRKY31-like2*, were expressed significantly lower at 24 hpi in Dusa® (Fig. 3). Similarly, *PaWRKY58* was significantly lower in Dusa® at 24 and 120 hpi. The expression of *PaWRKY55*, *PaWRKY56* and *PaWRKY66*, was significantly higher in Dusa® when compared to R0.12 at 12 hpi with *PaWRKY55* displaying comparatively higher expression in Dusa® at 120 hpi as well.

When either rootstock was compared to its respective uninoculated control, only three *PaWRKY-like* genes were significantly up- or downregulated at any of the earlier time points (Fig. 2). These three genes, *PaWRKY16, PaWRKY26-like2* and *PaWRKY26-like3*, were identified as orthologs of *AtWRKY53*. *PaWRKY16*, and were significantly downregulated at 6 and 12 hpi in Dusa®, followed by a return to baseline at 24 hpi and significant upregulation at 120 hpi. Meanwhile, in R0.12 *PaWRKY16* was significantly downregulated at 6 hpi only and upregulated significantly at 120 hpi. When comparing the expression of *PaWRKY16* between rootstocks, the only significant difference observed was at 120 hpi, where expression was significantly higher in Dusa® (Fig. 3). In both rootstocks *PaWRKY26-like2* was significantly upregulated at 6 hpi followed by non-significant differences in expression at all other time points when compared to their respective uninoculated controls (Fig. 2). However, when comparing the expression between rootstocks, *PaWRKY26-like2* was significantly lower in Dusa® at 12 hpi (Fig. 3). By comparison, *PaWRKY26-like3* was significantly upregulated at 6, 24 and 120 hpi when comparing Dusa® to its uninoculated control, but only at 6 hpi in R0.12. Additionally, the expression of this gene was substantially higher in Dusa® at 120 hpi when compared to R0.12.

Four putative orthologs of *AtTGA2* and two for *AtTGA4* were identified in *P. americana*. Interestingly, none of the *AtTGA2* like putative orthologs showed significant up- or downregulation when R0.12 was compared to its uninoculated control (Fig. 2). In Dusa®, minor yet significant downregulation was observed for *PaTGA2-like1* at 12 and 24 hpi, *PaTGA2-like2* displayed significant upregulation at 120 hpi while *PaTGA2-like3* was substantially downregulated at that time. The expression of *PaTGA2-like1* and *PaTGA2-like2* was significantly lower in Dusa® when compared to R0.12 at 12 and 24 hpi (Fig. 3). While the expression of *PaTGA-like3* tended far lower when comparing Dusa® and R0.12 at 120 hpi, the difference did not attain statistical significance (Table S5). Notably however, the expression of *PaTGA2-like4* was significantly lower in Dusa® at 12 hpi when comparing the rootstocks. One of the identified *AtTGA4* orthologs, *PaTGA4-like1*, was significantly upregulated at 6 and 12 hpi in Dusa®, and at 12 hpi in R0.12 when compared to the uninoculated control samples. Similarly, *PaTGA4-like2* was significantly upregulated at 6, 12 and 120 hpi in Dusa®, while in R0.12 this gene was only upregulated at 120 hpi (Fig. 2). Nonetheless, no significant differences were observed for either of these genes when comparing any of the sample libraries between Dusa® and R0.12.

#### SAR-related genes

The expression of several PR protein families are dependent on TGA transcription factors and are vital to the establishment of SAR [17]. We identified three *AtPR1-like* and 17 *AtPR2-like* putative orthologs in *P. americana*, as well as one *AtPR5-like* ortholog. Of these, all *PaPR1-like* genes, nine *PaPR2-like* genes and one *PaPR5-like* gene were differentially regulated after inoculation with *P. cinnamomi*, when considering both rootstocks (Fig. 2). All three *PaPR1-like* genes, *PaPRB1-3-like1*, *PaPRB1-3-like2* and *PaPRB1-3-like3*, were significantly upregulated in Dusa® at 120 hpi, while only *PaPRB1-3-like3* reached significance in R0.12. Both *PaPRB1-3-like2* and *PaPRB1-3-like3* tended toward downregulation, in both rootstocks, at the earlier time points although only *PaPRB1-3-like3* in Dusa® reached significance at 24 hpi. When comparing rootstocks, both *PaPRB1-3-like2* and *PaPRB1-3-like3* appeared to be lower in Dusa® when comparing most samples to their counterparts in R0.12 (Fig. 3). However, there was significantly less *PaPRB1-3-like2* expression in the uninoculated control and 120 hpi samples as well as significantly less *PaPRB1-3-like3* expression in the 12 and 24 hpi samples from Dusa®.

Like *PaPR1-like* genes, significant upregulation of the *PaPR2-like* genes was largely observed at 120 hpi, with seven and five conforming to this observation in Dusa® and R0.12, respectively (Fig. 2). There were however several noteworthy exceptions, the first being *PaPR2-like2* which showed significant upregulation at all time points in Dusa®, and none in R0.12. *PaPR2-like3* was significantly upregulated at 6 and 12 hpi in Dusa® and at 6 hpi in R0.12. Meanwhile, *PaPR2-like8* and *PaPR2-like12* were significantly upregulated at 6 hpi in R0.12, but not Dusa®. Conversely, *PaPR2-like11* was significantly downregulated in Dusa® at 6 hpi, but not R0.12. Most of the differences noted in the expression of *PaPR2-like* genes when comparing Dusa® and R0.12 occurred at 6 and 12 hpi (Fig. 3). The abundance of *PaPR2-like5* in Dusa® was significantly lower at 6 and 12 hpi, while *PaPR2-like11* and *PaPR2-like12* were significantly lower at 6 hpi. Meanwhile the expression of *PaPR2-like8* was significantly upregulated in Dusa®, as compared to R0.12, when considering the uninoculated control and 12 hpi samples. Lastly, the expression of *PaPR5* was significantly downregulated at all time points in Dusa® and at 6 and 12 hpi in R0.12, when compared to their respective controls samples (Fig. 2). Thus, it was unsurprising to find that when comparing Dusa® to R0.12, significant downregulation of *PaPR5* was observed 12 and 24 hpi (Fig. 3).

Additionally, the expression of *A. thaliana* histone deacetylase *HDAC19* putative ortholog *PaHDAC1* was slightly, yet significantly downregulated at 12 hpi in Dusa® and not R0.12 when each were compared to their respective uninoculated sample libraries (Fig. 2). The expression of *PaHDAC1* was downregulated in Dusa® at 12 and 24 hpi when compared to the complementary sample libraries in R0.12 (Fig. 3). We also identified two *AtICS1-like* orthologs in *P. americana*, *PaICS-like1* and *PaICS-like2*. In Dusa®, the expression of *PaICS-like1* was slightly, but significantly downregulated at 6 and 12 hpi, while *PaICS-like2* was substantially downregulated at 120 hpi (Fig. 2). In R0.12 *PaICS-like2* was also significantly downregulated, slightly at 6 hpi and substantially at 120 hpi. Interestingly, the expression of *PaICS-like2* was significantly higher in Dusa® at 120 hpi, when compared to R0.12 (Fig. 3). Conversely, the *P. americana* putative ortholog of *AtSARD1*, *PaSARD1* was significantly upregulated at 120 hpi in Dusa® when compared to its uninoculated control, but not in R0.12.

Three *AtMPK3/6* orthologs were identified in *P. americana,* namely, *PaMMK1-like1, PaMMK1-like2,* and *PaMPK3*. Interestingly, the expression of neither of the *PaMMK-like* genes met the significance or log_2_FC cutoffs. However, *PaMPK3* was significantly differentially expressed in both Dusa® and R0.12 when compared to the uninoculated sample libraries (Fig. 2). In Dusa® *PaMPK3* was significantly upregulated at 6, 24 and 120 hpi, while in R0.12 this gene was significantly upregulated at 6 and 120 hpi. When comparing the rootstocks, significant upregulation of *PaMPK3* was observed in Dusa® at 12 and 24 hpi (Fig. 3).

#### ER-related genes

We further characterized several members of the protein secretory pathway, and the related transcription factor *AtHSFB1*, in *P. americana* based on their notable roles in the NPR1-dependent defense response pathway [17]. Six *AtHSFB1-like*, two *AtBiP2-like* and two *AtCRT3-like* orthologs were identified in *P. americana*. In Dusa® all *AtHSFB1-like* orthologs, except *PaHSFA3*, were significantly upregulated at 120 hpi when compared to control (Fig. 2). Two of these, *PaHSF24-like1* and *PaHSFB2b* were significantly downregulated at the early time points, 6, 12 and 24 hpi in Dusa®. However, in R0.12 *PaHSF24-like1* was only significantly downregulated at 6 and 12 hpi, with no up- or down regulation at any other time point. Similarly, *PaHSFB2b* was only downregulated at 6 hpi in R0.12. *PaHSFB3-like1* and *PaHSF24-like2* were significantly upregulated in both rootstocks at 120 hpi. However, in Dusa® *PaHSF24-like2* was also significantly downregulated at 24 hpi. The last *AtHSFB1-like* ortholog, *PaHSFA3*, was significantly upregulated in R0.12 at 6 hpi. When comparing the rootstocks *PaHSFB2b* and *PaHSF24-like2* displayed significantly lower expression in Dusa® when compared to R0.12 (Fig. 3). *PaHSF24-like1* also displayed significant downregulation in Dusa, but only at 24 hpi.

*PaBiP4* showed no significant differential expression at any time point in Dusa®, however, it was significantly downregulated in R0.12 at 6 hpi (Fig. 2). The expression of *PaBiP5* in Dusa® was downregulated at 24 hpi and upregulated at 120 hpi, although only slightly within the cutoff set for this study. There was also a slight yet significant downregulation of *PaBiP5* in Dusa® at 24 hpi when compared against R0.12 (Fig. 3). In Dusa®, *PaCRT3-like1* was significantly downregulated at 6, 12 and 24 hpi followed by a return to baseline at 120 hpi (Fig. 2). The expression of this gene was only significantly downregulated in R0.12 at 6 hpi. Suprisingly, *PaCRT3-like2* was significantly upregulated in Dusa® at 6 hpi and downregulated in R0.12 at 120 hpi. When observing for differences between rootstocks it was noted that *PaCRT3-like1* was significantly downregulated in Dusa® when compared to R0.12, at 12 and 24 hpi (Fig. 3). Another member of the protein secretory pathway in *P. americana*, *PaSec61α-like2*, was similarly downregulated in Dusa® at 12 and 24 hpi, when compared to R0.12. Interestingly, neither of the putative orthologs for *AtDAD1* in *P. americana* were expressed to an appreciable degree.

#### Others

Finally, we describe orthologs for the SA-responsive gene, *AtTOC1*, and its antagonist *AtLHY* in *P. americana*. The expression of *PaTOC1-like1* is significantly upregulated at 6 and 12 hpi in both rootstocks (Fig. 2). Interestingly expression returns to almost exact control-like levels at both 24 hpi and 120 hpi. In contrast, *PaLHY-like2* expression contrasts the expression of *PaTOC1-like1*, displaying significant downregulation at 6 and 12 hpi in both rootstocks followed by a swift return to baseline levels at 24 and 120 hpi. However, another ortholog of *AtLHY*, *PaLHY-like1* is significantly downregulated in R0.12 at 12 hpi while being slightly, yet significantly upregulated in Dusa® at 24 hpi. Comparison between Dusa® and R0.12 revealed that the expression of *PaLHY-like1* is significantly higher in the former at 12 hpi (Fig. 3).

## Discussion

This study explored the expression of NPR1 pathway-associated genes, over time, in both *P. cinnamomi* susceptible (R0.12) and partially resistant (Dusa®) *P. americana* rootstocks, following inoculation. Expression was also compared between R0.12 and Dusa®, allowing for more direct comparisons during each of the sampled conditions. The observations presented here confirmed the hypotheses set forth for this study. Notably, 64 out of 92 identified NPR1 pathway-associated genes were responsive to inoculation with *P. cinnamomi*, in Dusa®. Meanwhile, only 51 were responsive to the same stimulus in R0.12. These genes conform, mostly, with our expectation based on the literature, representing an intact and responsive SA-induced NPR1-dependent pathway. For instance, significant upregulation of several *PR-like* genes at 120 hpi indicated the establishment of SAR in both rootstocks. However, several differences were evident when comparing R0.12 and Dusa®, most markedly at 12 and 24 hpi. Overall, the evidence presented here suggests that Dusa® maintains a robust NPR1-dependent defense response pathway during the early stages of *P. cinnamomi* challenge. Conversely, R0.12 only displays comparable patterns of gene regulation at 6 and 120 hpi. Thus, it is likely that the differences in *P. cinnamomi* sensitivity between R0.12 and Dusa® may be dependent on variations in the regulation of the NPR1 pathway during the early stages of infection.

### Challenge with *P. cinnamomi* leads to the establishment of SAR

SAR is initiated in response to threat from biotrophic and hemibiotrophic pathogens, inciting broad-spectrum resistance to additional impending pathogenic stresses [7]. Usually, the successful establishment of SAR is evidenced by a clear increase in the abundance of *PR-like* transcripts [5, 10, 11]. In this study, significant upregulation of three *PaPR1-like* and seven *PaPR2-like* genes in Dusa®, and one *PaPR1-like* and five *PaPR2-like* genes in R0.12 were noted by 120 hpi. Most importantly, the upregulation of these genes at 120 hpi was substantial in both rootstocks, with log_2_FC’s ranging from 1.76 to 4.62 in Dusa®, and 1.76 to 2.92 in R0.12. Furthermore, of the 92 genes investigated in this study six *PaPR-like* genes were among the top 10 with the highest average expression (baseMean), further underscoring their importance. Thus, by definition, we can confirm that SAR was established in both Dusa® and R0.12 in response to *P. cinnamomi* inoculation. And while we could have predicted such an outcome for Dusa®, a similar expectation of R0.12 was more tenuous. Nonetheless, it is not incomprehensible that some shared molecular defense characteristics would be present in both rootstocks, especially given that R0.12 was initially classified as tolerant to *P. cinnamomi* [3].

Interestingly, when interrogating the comparative expression data for Dusa® and R0.12, *PaPRB1-3-like2*, *PaPRB1-3-like3*, *PaPR2-like5*, *PaPR2-like11* and *PaPR2-like12* were expressed significantly lower in Dusa® at various time points. When viewed alongside the time course data, these observations suggest that the proteins encoded by these genes likely were not contributing to the enhanced *P. cinnamomi* resistance in Dusa®, even though they were induced during SAR. Contrastingly, *PaPR2-like8* was not significantly regulated in Dusa® at any time in response to *P. cinnamomi* (Fig. 2). However, significantly more *PaPR2-like8* transcripts were present in Dusa® at baseline and 12 hpi, when compared to R0.12 (Fig. 3). Thus, *PaPR2-like8* overexpression or knock-out studies would be an attractive avenue of exploration to determine whether increased basal expression of *PaPR2-like8* contributes to enhanced *P. cinnamomi* resistance in Dusa®.

Another family of PR proteins, PR5, have been shown to be effective antifungal agents, both in vitro and in vivo [99–102]. Moreover, constitutive overexpression of a tomato *PR5* in orange plants decreased susceptibility to *Phytophthora citrophthora* [103]. In the present study *PaPR5* was significantly downregulated at all time points in Dusa® and at 6 and 120 hpi in R0.12. This observation was surprising, especially given the magnitude of the downregulation evidenced. Furthermore, when comparing Dusa® and R0.12 directly, *PaPR5* expression was significantly lower in Dusa® at 12 and 24 hpi. Thus, our observations suggest that *PaPR5* is likely not involved in the defense against *P. cinnamomi* and that *PaPR5* does not contribute to the establishment of SAR.

The accumulation of *MPK3* mRNA and inactive proteins are required for SAR, and vital for SA-dependent defense gene priming in preparation for future biotic stress [62]. The upregulation noted for *PaMPK3* was thus to be expected following inoculation with *P. cinnamomi*. Interestingly, in both Dusa® and R0.12, *PaMPK3* was upregulated at both the earliest and latest time points; indicating that NPR1-dependent priming occurs in *P. americana*, another hallmark of successful SAR induction. When comparing expression between the rootstocks we again noted significant differences at 12 and 24 hpi, with Dusa® displaying significantly more *PaMPK3* expression at these times. Thus, it seems that *PaMPK3* is important during both the early signaling events in *P. americana,* as well as SAR and the priming of SA- and NPR1-dependant defense genes; a conclusion which is concordant with previous findings [62–64].

### Activation of the NPR1-dependent defense response pathway

Several genes which encode for secretory pathway proteins are known to be essential for optimal PR protein secretion following the induction of SAR [55]. Additionally, the transcription factor AtHSFB1 has been implicated in the transition from growth to defense and the expression of several endoplasmic reticulum (ER)-associated protein coding genes, including *LUMINAL BINDING PROTEIN 2* (*BiP2*)*, DEFENDER AGAINST APOPTOTIC DEATH 1 (DAD1*)*, Sec61α* and *CALRETICULIN 3* (*CRT3*) [34]. Therefore, we assigned value to the inclusion of the *P. americana* putative orthologs for these genes in the current study. We noted that several orthologs of *AtHSFB1* were upregulated in tandem with *PaPR-like* genes at 120 hpi, in both Dusa® and R0.12. However, the number of upregulated orthologs was higher in Dusa® when considering only the time-course data. The ortholog of *AtBiP2*—*PaBiP5—*was also significantly upregulated at 120 hpi in Dusa® but not R0.12. Nonetheless, the comparison data for Dusa® and R0.12 indicated that no significant differences were observed for any of the identified ER-associated protein-coding genes or *AtHSFB1* orthologs at 120 hpi. Several of these and other ER-associated protein coding genes were significantly downregulated in Dusa® at 12 and 24 hpi, when compared to R0.12. These data indicate that the response in both rootstocks was comparable during the late stages of *P. cinnamomi* infection, but that differences reside during the earlier 12 and 24 hpi time points. Of note, upregulation of ER-associated genes may occur at earlier time points than those included in this study. In *Arabidopsis*, *AtHSFB1, AtCRT3* and *AtBiP2* were upregulated within 4 h and returned to baseline levels within 8 h of SA treatment [34], thus future investigations in *P. americana* might consider the inclusion of additional early time points. Nonetheless, both *P. americana AtHSFB1-like* orthologs and related secretory pathway genes were regulated in response to *P. cinnamomi* inoculation. Thus, further characterization of their roles in defense against *P. cinnamomi* could prove invaluable.

The expression of *AtHSFB1* and *AtNPR1* have been suggested to be codependent [34]. In the current study the data tend to suggest a similar codependency, as the trend for expression of *PaHSFB3-like1* and *PaHSFB3-like2* mimic that of *PaNPR1* and *PaNPR2*. In both Dusa® and R0.12, upregulation of *PaNPR1* and *PaNPR2* occurs at 120 hpi. Furthermore, no significant differences in the expression of *PaNPR1* and *PaNPR2* were noted between the two rootstocks. Similar observations were made following inoculation of grapevine with *Plasmopara viticola*, a biotrophic oomycete [104]. The authors of this study noted no transcriptional level changes for *VvNPR1.1* or *VvNPR1.2* expression in either susceptible (*Vitis vinifera*) or resistant grapevine species (*V. riparia*), attributing enhanced resistance to protein level regulation. Such conclusions for both *P. americana* and *Vitis* spp. are not inconceivable as it has long been believed that the regulation of NPR1 occurs predominantly at the post-translational level [105]. However, transcriptional regulation of NPR1 may occur at time points much earlier than were investigated in this study. In another study on grapevine, upregulation of *NPR1* occurred within 2 h of *P. viticola* inoculation and for most of the tested species and cultivars, expression declined by 6 hpi [106]. Thus, whether upregulation of *PaNPR1* or *PaNPR2* occurs at unsampled time points can not be ruled out and should be accounted for in future studies. However, characterizing additional factors which contribute to the activity of NPR1 at the protein level should be considered in tandem.

Perhaps the most well documented protein level regulatory mechanism for NPR1 involves the transition from the inactive oligomeric form to the active monomeric form; which is, at least in part, controlled by TRXs, GSNO and GSNOR [38, 39, 41–43, 47, 107]. However, several studies have indicated that the shift from oligomer to monomer following SA application, or biotic stress, is not universally conserved across plant species [38, 56, 104]. Even so, the downregulation of *PaTRXs* in Dusa® and R0.12 in our time course data was surprising, given that we expected upregulation of these genes at the earliest time points. Furthermore, *PaTRX*s were significantly downregulated in Dusa® when considering the comparison to R0.12 at 12 and 24 hpi. Previously, *AtTRX5* was shown to be induced following exposure to abiotic and biotic stressors [108, 109]. And while *AtTRX3* remained constitutively expressed following pathogenic elicitor treatments, both *AtTRX5* and *AtTRX3* were shown to be essential for complete induction of *AtPR1* following SA treatment [39]. We also failed to observe significant upregulation of *PaADH3* at any time point. Thus, our evidence suggests that TRXs, GSNO and ADH3/GSNOR may not be prominent factors in the *P. cinnamomi* partial resistance phenotype seen in Dusa®. The data also suggest that the activity of PaNPR1 and PaNPR2 are likely not regulated by the oligomer to monomer transition. Instead, these proteins are likely to exist as monomers within the nucleus of *P. americana* cells in which SA concentration has not deviated from baseline; a possibility not addressed in the current study but adequately supported by literature [38, 56, 104].

In addition to monomerization, protein phosphorylation has been shown to be essential for NPR1 to function appropriately during the induction of SAR [45, 46, 50]. In tissues distal to the initial site of infection where SA concentration is lower, SRK2C phosphorylates NPR1 precipitating its’ nuclear import during the onset of SAR [45]. Given that the samples included in this study were comprised of full-length root tissue, it would be reasonable to assume that gene expression represents both local and distal defense responses. The significant upregulation of several SRK2C orthologs, primarily at 12 hpi, in both R0.12 and Dusa® would imply an increased potential for phosphorylation and subsequent activation PaNPR1 and/or PaNPR2. Notably, *PaSRK2-like5* was significantly upregulated in Dusa® when compared to R0.12 at 12 hpi, warranting consideration of this gene for some of the differences seen between these rootstocks.

Furthermore, the kinase AtCIPK11 has been shown to interact with NPR1 and phosphorylate its C-terminal region [46]. In so doing, CIPK11 positively regulates the expression of *AtWRKY38* and *AtWRKY62*, two negative regulators of the SA-signaling pathway [28, 46]. How exactly the phosphorylation of the NPR1 C-terminal domain leads to increased *WRKY* expression remains to be determined. Nonetheless, the *AtCIPK11* ortholog, *PaCIPK10-like1*, was significantly upregulated at 120 hpi in both *P. americana* rootstocks, corresponding with the upregulation of several *PaWRKY-like* genes. Meanwhile, downregulation of both *PaCIPK10-like* genes was noted promptly following *P. cinnamomi* inoculation. Most prominently, when comparing the expression of *PaCIPK10-like* genes between rootstocks, expression was decidedly lower in Dusa®, especially at 12 and 24 hpi, where SA-mediated defenses are expected to dominate [110]. Thus, downregulation of these kinases during the initial stages of infection, particularly in Dusa®, may limit negative regulation of the SA-signaling pathway early-on. In addition, we surmise that PaCIPK10-like1 might lead to upregulation of select *PaWRKY-like* genes through phosphorylation of PaNPR1 and/or PaNPR2; in so doing, PaCIPK10-like1 would indirectly limit perpetual and inappropriate SA-signaling during the necrotrophic phase of *P. cinnamomi*’s life cycle.

Phosphorylation of various NPR1 residues also target it for CUL3 E3 ligase-facilitated ubiquitinylation and subsequent degradation by the 26S proteasome [49, 50]. Paradoxically, the degradation of NPR1 is required for the development of SAR, an observation attributed to the turnover of spent proteins [50]. Concomitantly, sumoylation of NPR1 by SUMO3 leads to increased NPR1 phosphorylation [49]. This, in turn, increases the expression of SAR related genes by decreasing NPR1’s association with WRKYs, increasing proteasome-mediated turnover, and increasing association with TGAs [49]. Although we noted slight decreases in the expression of *PaSUMO2* in both Dusa® and R0.12, the expression of *PaCUL3A-like* genes was significantly increased within the first 24 h of *P. cinnamomi* challenge. And interestingly, at 12 and 24 hpi significantly more *PaCUL3A-like2* transcripts were present in Dusa® as compared to R0.12. This observation might indicate that the necessity for turnover of spent PaNPR-like proteins is higher in Dusa®. Thus, there is mounting evidence for the induction of a stronger and more robust SA- and NPR1-dependent signaling event in Dusa® following *P. cinnamomi* inoculation. Nonetheless, several differences were observed between most genes putatively involved in the post-translational modification of PaNPR1 and PaNPR2. Together, these observations suggest that post-translational modification may play a role in the increased resistance of Dusa® during *P. cinnamomi* challenge.

Even though we have established that PaNPR1 and PaNPR2 likely exist in an active state within the nucleus soon after *P. cinnamomi* challenge, we have yet to consider the role of interacting proteins which alter NPR1 activity. The NIMINs are one such group, and as negative regulators their overexpression compromises SAR induction and the expression of *PR* genes [57, 111]. From our data we showed that one *AtNIMIN-like* ortholog, *PaNIMIN2-like4*, was significantly downregulated in both Dusa® and R0.12; however, some notable differences were observed in its expression, both over time and between rootstocks. This further highlights that the regulation of the NPR1-defense response pathway diverges substantially between rootstocks, especially at 12 and 24 hpi. While these differences are substantial, it should be noted that NIMINs do not prevent SAR-related gene expression outright, though they may delay it through differing sensitivities to SA [58]. Thus, it is likely that the expression of *PaNIMIN2-like4* affects the temporal milieu of *PaPR-like* gene expression between Dusa® and R0.12.

Further members of the NPR-like family, NPR3 and NPR4, have also been shown to interact with NPR1 [112–114]. Intriguing models have been proposed regarding their opposing effects on NPR1-dependent gene expression in the presence of differing SA concentrations [19, 53]. The first of these proposed that, in tissues proximate to pathogen infiltration where SA concentration is highest, NPR3 facilitates the rapid the degradation of NPR1 to limit its documented HR suppressing activity [53, 115]. The upregulation of *PaNPR4*, especially in Dusa®, coheres with this model. Being a hemibiotrophic pathogen, *P. cinnamomi* is believed to exists in a biotrophic state during the first 12–18 hpi when interacting with *P. americana* [110]. During this time, the HR should effectively limit further pathogen infiltration and the increased abundance of *PaNPR4* may assist in this outcome. Given that significantly less *PaNPR4* was present in R0.12 at 12 and 24 hpi, it is tempting to postulate that this rootstock may not initiate the HR effectively, increasing its susceptibility to *P. cinnamomi* early-on.

Several transcription factors, particularly WRKYs and TGAs, have also been shown to directly interact with NPR1 to control the expression of SAR-related genes [21, 24, 25, 49, 116]. Interestingly, the majority of WRKY transcription factors are linked to pathogen responses and SA-signaling [117–119]. However, their collective role is complex, including both negative and positive regulatory effects on the SA-signaling pathway [27–31]. Even so, negative regulators of SA-signaling are essential for the complete induction of SAR. To that end, it was demonstrated that both WRKY38 and WRKY62 are crucial to the induction of SAR in *A. thaliana* in an NPR1-dependent fashion [50]. Unsurprisingly, the majority of *WRKY-like* orthologs in *P. americana* were significantly upregulated at 120 hpi in both Dusa® and R0.12. Additionally, the expression of *PaICS1-like2* was downregulated significantly at 120 hpi. Together these observations indicated that the SA-signaling pathway was being suppressed to some extent by *PaWRKYs*, at 120 hpi in both rootstocks. There were also apparent differences, with *PaWRKY*s being upregulated in one rootstock but not the other. Furthermore, the comparative data indicated a stronger global induction of *PaWRKY*s in Dusa®. Therefore, it seems that the SA-signaling pathway was more definitively suppressed at 120 hpi in Dusa®, given the general negative regulatory role WRKYs have on SA-signaling. Although most *PaWRKYs* were not downregulated significantly during the early stages of infection, most tended toward decreased expression, likely to prevent suppression of SA-signaling events early-on. The exceptions to this general observation are *PaWRKY26-like2* and *PaWRKY26-like3*, both of which displayed increased expression early-on following *P. cinnamomi* inoculation. The proteins encoded for by these genes might play a role in positive regualtion of the SA-signalling pathway.

Similarly, two *AtTGA-like* orthologs, *PaTGA4-like1* and *PaTGA4-like2*, were significantly upregulated soon after *P. cinnamomi* inoculation. We can thus safely assume that these *PaTGA*s might be involved in the expression of SA-induced genes in *P. americana*. Conversely, *PaTGA2-like* genes tended toward downregulation during the initial stages of infection in both rootstocks. However, significantly less *PaTGA2-like* gene expression was noted in Dusa® when comparing both rootstocks. These results indicate that expression of these genes may negatively impact SA-mediated defense responses, as previously reported [24, 26]. Alternatively, they may have another role altogether, as TGAs have been implicated in everything from developmental processes to circadian rhythm [120].

Both *PaNPR3* and *PaNPR5*, were previously suspected of fulfilling a role in growth and development based on their shared structural similarity and evolutionary relationship with *AtBOP1* and *AtBOP2* [79]. These observations were further supported by the downregulation of *PaNPR5* in the current study. Downregulation of *PaNPR5* was significant in both Dusa® and R0.12 at 6 hpi and persisted in Dusa® at 12 and 24 hpi, but not R0.12. Moreover, previous analyses of the data utilized in this study indicated that genes related to growth and developmental pathways were downregulated at 12 and 24 hpi in Dusa®, but not R0.12 [98]. This not only adds support to the putative developmental role of *PaNPR5* but to the disparity in the duration of the initial defense responses between Dusa® and R0.12, as growth and defense are generally considered antithetical processes.

## Conclusion

This study represents the first comprehensive investigation into the role of the NPR1 pathway during *P. cinnamomi* challenge in *P. americana*. We demonstrated the establishment of SAR in response to *P. cinnamomi* inoculation. We further described the most likely mechanisms employed to achieve SAR, with a focus on NPR1 and associated pathways. Significant differences in the regulation of putative pathway genes were observed when comparing susceptible and partially resistant *P. americana*-*P. cinnamomi* interactions. Overall, the evidence presented here suggests that the SA- and NPR1-dependent defense response pathways were active for at least the first 24 h following *P. cinnamomi* inoculation in Dusa^®^. By contrast, there was no clear evidence for the activation of these pathways at 12 and 24 hpi in R0.12. Additionally, while several investigated SA-signaling pathway genes suggested that both Dusa^®^ and R0.12 initiated SAR by 120 hpi, Dusa^®^ seemed to upregulate several suppressive components, such as *PaWRKY*s and *PaNIMIN2-like4,* by 120 hpi. Together, these observations indicated that the combination of a prolonged SA- and NPR1-dependent defense response during *P. cinnamomi*’s biotrophic phase, and more definitive suppression of these pathways following the onset of necrotrophy, may be at least partly responsible for the increased resistance to *P. cinnamomi* in Dusa^®^. While this study represents a substantial gain in understanding the role of the extended NPR1 pathway in defense against *P. cinnamomi*, it also highlights some important questions. To that effect, it would be worth investigating, to a similar degree as presented here, the regulation of SA- and NPR1-dependant gene expression in additional rootstocks of varying susceptibilities during *P. cinnamomi* challenge. Additionally, while the evidence presented here might highlight some critical aspects of NPR1-dependent defense responses in avocado, further molecular investigations would be needed to prove any conclusively.

### Supplementary Information

**Additional file 1: Table S1.** Plant proteomes obtained as input for orthologue identification using OrthoFinder v2.5.4. **Table S2.** Candidate NPR1 pathway-associated genes in *Persea americana*. Putative functional descriptions are the result of a combined annotation approach by using original functional annotations (Avocado Genome Consortium) and eggNOG-mapper, InterProScan and BLASTp descriptions. Candidates with no expression data, determined using baseMean following DeSeq2 analyses, were not assigned descriptors. **Table S3.** Protein-protein BLAST of identified *Persea americana* NPR1-dependent defense response pathway proteins. The NCBI non-redundant protein sequences database was used and limited by entrez entry Viridiplantae with an Expected (E) value cutoff 0.05, word size 3, BLOSUM 62, Gap cost (existence 11, extension 1), max hits per sequence 5. **Table S4.** Differentially expressed *Persea americana* NPR1- pathway-associated genes (log2(fold change; log2FC) > 0.58 | < -0.58, adjusted p-value (FDR; p-adj) < 0.05) at various time-points following inoculation of the partially resistant rootstock Dusa^®^ and the susceptible rootstock R0.12 with *Phytophthora cinnamomi*, compared to a mock-inoculated control. lfcSE - standard error of log2FC.** Table S5.** Differentially expressed *Persea americana* NPR1- pathway-associated genes (log2(fold change; log2FC) > 0.58 | < -0.58, adjusted p-value (FDR; p-adj) < 0.05) comparing the expression of uninoculated (Control) sample libraries or various *Phytophthora cinnamomi* inoculated libraries, of the partially resistant rootstock Dusa^®^ with the respective libraries in the susceptible rootstock R0.12. lfcSE - standard error of log2FC. 

## Data Availability

The raw datasets supporting the conclusions of this article are available in the Sequence Read Archive of NCBI Genbank repository, PRJNA675400 (https://www.ncbi.nlm.nih.gov/bioproject/PRJNA675400/). In addition, the processed datasets supporting the conclusions of this article are included within the article (and its additional file(s)).
